# Effects of air pollution exposure on social behavior: a synthesis and call for research

**DOI:** 10.1186/s12940-021-00761-8

**Published:** 2021-06-25

**Authors:** Chelsea A. Weitekamp, Hans A. Hofmann

**Affiliations:** 1 Center for Public Health and Environmental Assessment, U.S. Environmental Protection Agency, Durham, NC USA; 2 Department of Integrative Biology, The University of Texas At Austin, Austin, TX USA; 3 Institute for Cellular and Molecular Biology, The University of Texas At Austin, Austin, TX USA; 4 Institute for Neuroscience, The University of Texas At Austin, Austin, TX USA

**Keywords:** Air pollution, Social behavior, Particulate matter, Ozone, Social decision-making network, Dopamine, Sex steroids, Inhalation, Hazardous air pollutants

## Abstract

**Background:**

There is a growing literature from both epidemiologic and experimental animal studies suggesting that exposure to air pollution can lead to neurodevelopmental and neuropsychiatric disorders. Here, we suggest that effects of air pollutant exposure on the brain may be even broader, with the potential to affect social decision-making in general.

**Methods:**

We discuss how the neurobiological substrates of social behavior are vulnerable to air pollution, then briefly present studies that examine the effects of air pollutant exposure on social behavior-related outcomes.

**Results:**

Few experimental studies have investigated the effects of air pollution on social behavior and those that have focus on standard laboratory tests in rodent model systems. Nonetheless, there is sufficient evidence to support a critical need for more research.

**Conclusion:**

For future research, we suggest a comparative approach that utilizes diverse model systems to probe the effects of air pollution on a wider range of social behaviors, brain regions, and neurochemical pathways.

## Background

Ambient air pollution is ubiquitous and impacts individuals across ages and all geographic regions, but also often entails inequities in exposure, thereby exacerbating socioeconomic and racial-ethnic disparities [1]. Its burden on public health is substantial, driven in part by urban development, globalized industrial production, and use of motor vehicles with internal combustion engines [2]. While it has been well-established that long-term exposure to ambient air pollution increases risk of morbidity and mortality from cardiovascular and respiratory disease [3], there is a growing literature showing that air pollutant exposure also impacts neural function and social-neurobehavioral outcomes in humans [4–6], such as autism spectrum disorder [7–11], bipolar disorder [12], and depression [13]. Experimental studies in animal models have supported the epidemiologic evidence, showing that in rodents, inhalation of air pollutants can lead to characteristics of neurodevelopmental and neuropsychiatric disorders [14–16]. Therefore, if exposure to air pollution can affect the incidence or severity of these disorders, even at ambient levels, how might air pollutants affect social behavior more generally? We believe this is an important question with the potential to have both societal consequences for humans and implications for wildlife. In humans, air pollution has been associated with reduced social competence (i.e., ability to effectively handle social interactions) [17] and increased violent behavior [18, 19], but there is a notable absence of experimental research on the effects of air pollutant exposure on social decision-making and social behavior more broadly.

Social behavior is defined as interactions among two or more organisms where one individual affects the other, usually within the same species [20]. These behaviors can include collective behaviors such as swarming, schooling, and flocking, as well as (usually) dyadic behaviors such as parental care, pair bonding, aggressive behavior, cooperation, mate choice, and sexual behavior. Given the prominent role of hormones in mediating behavior, the effects of other environmental contaminants such as endocrine disrupting chemicals (e.g., those that interfere with hormones and their actions) have been studied in diverse taxa since the 1970s [21–23]. This research has lent insight into the dramatic ways in which chemical exposures in the environment can modify social behaviors, particularly when exposures occur during susceptible periods of development [22]. While oral and dermal sources of endocrine disrupting chemicals have been most extensively studied, another important route of exposure is through inhalation of outdoor air [24, 25]. For neural effects, inhalation is a unique route of exposure because compounds can bypass the blood–brain barrier and reach the brain directly. In the air, endocrine disrupting chemicals exist both as gases and as components of particulate matter [25]. Yet air pollution is a complex mixture and potential effects on social behavior from exposure can arise from mechanisms beyond endocrine disruption, including oxidative stress and neuroinflammation, as well as direct neuronal damage [26]. These effects can occur following exposure during development, which can lead to perturbations in brain development and subsequently alterations in adult social behavior, or from exposure in adult animals.

Air pollution is derived from both natural and anthropogenic sources. The U.S. EPA groups air pollutants into two major classes based on the way in which they are regulated: criteria air pollutants and air toxics. The six criteria air pollutants are broadly found across the U.S., have multiple sources, and may reasonably be anticipated to endanger public health or welfare. These include ozone, particulate matter, lead, nitrogen dioxide, sulfur dioxide, and carbon monoxide. The Clean Air Act of 1963 governs the establishment, review, and revision of the National Ambient Air Quality Standards for each criteria air pollutant. In 1990, amendments to the Clean Air Act identified 189 air toxics, or hazardous air pollutants, and defined a process for regulating emissions of these air pollutants. The air toxics are pollutants that cause serious irreversible, or incapacitating reversible, health effects including cancer and other serious health outcomes. There are currently 187 air toxics identified under the Clean Air Act (following delisting of two pollutants), including, for example, carbonyls, dioxins, polycyclic organic matter, polychlorinated biphenyls, and metal compounds, among many others. While levels of many air pollutants have decreased in the U.S. due to regulations under the Clean Air Act, air pollution remains a global concern. In 2016, 91% of the world population was living in areas that exceeded the World Health Organization air quality guidelines [27].

### Air pollutant exposure affects the brain

**Fig. 1:**
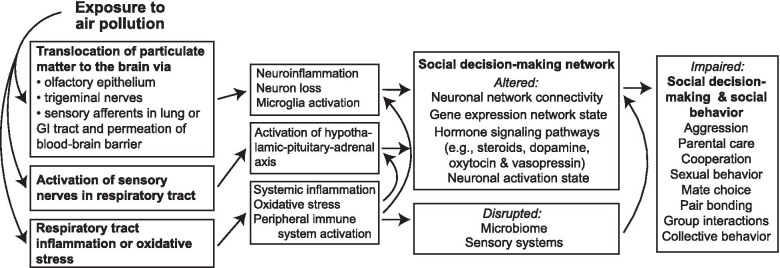
Potential pathways by which exposure to air pollution could affect social behavior

Air pollutants affect the central nervous system through both direct and indirect routes. Particulate matter is often cited as posing the greatest risk to health, which likely varies with particle size. This risk may arise from the particles themselves or from effects of the chemicals adsorbed onto the particles. Fine and ultrafine particulate matter (less than 2.5 and less than 0.1 µm in diameter, respectively) can deposit into the lungs, migrate into systemic circulation, and ultimately cross the blood–brain barrier [28–30]. Additional direct exposure routes include the potential for transport via the olfactory epithelium [31], as well as via sensory afferents in the gastrointestinal tract [4]. Once pollutants reach the brain, the innate immune system plays an important role in mediating neurotoxicity. Microglia, the resident immune cells in the brain, may attack pollutants, resulting in chronic or excessive activation and subsequent neuroinflammation through the release of pro-inflammatory cytokines and reactive oxygen species [32]. Microglia-induced neuroinflammation or oxidative stress can also occur in the absence of direct translocation to the brain [28, 33, 34]. Indeed, the dominant route of neuroimmune activation arising from air pollution exposure is likely via peripheral immune activation. For example, gaseous pollutants, such as ozone, can activate pulmonary macrophages, leading to a pro-inflammatory lung response that affects the central nervous system [34]. Therefore, air pollutant exposure can result in adverse neuronal effects via either direct or indirect contact with the brain (Fig. 1).

Notably, pollutants found in the air can also take the form of other exposure routes, such as ingestion of contaminated food or drinking water or dermal contact. For many of these pollutants, such as polychlorinated biphenyls [35], neurotoxicity and neuroendocrine disruption via oral exposure routes have been well-studied, but parallel studies that utilize inhalation exposure are lacking [36]. Inhalation of air pollutants is unique because it results in exposure to a complex mixture of compounds that can directly reach the brain, as described above.

## Methods

Our primary objective is to point to the biological plausibility for the effects of air pollution on a wide range of social behaviors in both human and non-human animals. As a basis for drawing conclusions, we briefly review the neural mechanisms of social behavior, focusing on the most relevant neural circuits and neurochemicals, and point to studies that show air pollutants can perturb these systems. Next, we discuss results from experimental studies that examined the effects from any air pollutant exposure on a social behavior-related outcome. To identify these experimental studies, we searched PubMed, Web of Science, and Google Scholar for social behavior-related search terms (social behavior, social interaction, parental care, paternal behavior, maternal behavior, reproductive behavior, sexual behavior, aggressive behavior, aggression, autism, or autistic) combined with one or more air pollutant search terms (particulate matter, ozone, air pollution, aerosol, vehicle emissions, diesel exhaust, nitrogen dioxide, sulfur dioxide, carbon monoxide, lead (with air or gaseous), inhalation exposure, or gaseous pollutants). We also reviewed reference lists from relevant and recently published research. Finally, given the evolutionary conservation of the mechanisms of social behavior, we point to five groups of non-rodent model systems that are well-suited to examine the effects of air pollutant exposure on a diversity of social behaviors and their mechanisms.

## Results

### Neurobiological substrates of social behavior are vulnerable to air pollutant exposure

The specific mechanisms through which air pollutants affect the brain, as well as the neural and molecular mechanisms through which social behavior arises are poorly understood. There are many layers of regulation between the brain and the expression of behavior, meaning there are just as many ways by which these systems can be perturbed by air pollution.

#### Evolutionarily conserved neural circuits

The expression of social behavior starts from the perception and evaluation of sensory cues, followed by an integration with the internal physiological state. This requires several layers of coordinated activity across cells in the brain. The first is via neuronal networks, which describes the transmission of electrochemical signals between neurons. An additional layer of activity occurs within gene regulatory networks, a collection of regulatory relationships among genes expressed within brain cells. The existence of gene regulatory networks was put forth following abundant evidence that gene expression profiles in the brain are associated with specific behavioral responses [37]. For social behavior, gene regulatory networks are likely unique given the addition of the social network component to information processing [37]. While spatial and temporal coordination occurs across the brain, there are 14 brain regions that have been well-studied for their role in social behavior (Table 1), most of which comprise the social decision-making network. In vertebrates, the social decision-making network is a highly conserved fore-/midbrain neural circuit that contains mesolimbic and hypothalamic brain regions [20, 38]. Within the brain regions of the social decision-making network, neuromodulatory systems such as steroid hormones, neuropeptides, and monoamines further integrate neural signals to result in the expression of social behavior.

**Table 1:** Brain regions of the social decision-making network and their primary known functions in social behavior [39]

medial amygdala	meAMY	aggression, reproduction, parental care, social recognition
medial bed nucleus of the stria terminalis	BNST	motivation, parental care, reproduction, stress response
preoptic area	POA	aggression, reproduction, parental care
lateral septum	LS	emotional learning, social affiliation/recognition, reproduction, parental care
ventromedial hypothalamus	VH	aggression, reproduction, parental care
anterior hypothalamus	AH	aggression, reproduction, parental care
midbrain periaqueductal gray	PAG	reproduction, vocalization
hippocampus	HIP	spatial learning
basolateral amygdala	blAMY	aggression, emotional learning, parental care
ventral tegmental area	VTA	motivation, reproduction, parental care
striatum	Str	compulsive behavior
nucleus accumbens	NAcc	emotional learning, impulsivity, motivation, parental care
ventral pallidum	VP	emotional learning, parental care
cerebral cortex	CC	integration, decision-making

*Potential link to air pollution exposure*: Air pollution-induced changes in social behavior could be driven by neuroinflammation in regions of the social decision-making network [26, 40]. Interestingly, neuroinflammation is closely associated with the dysregulation of this network in mood disorders and suicide mortality [41] and substance use disorders [42]. Related to this, neuroinflammation arising from peripherally produced pro-inflammatory cytokines can result in ‘sickness behavior’, an adaptive response to immune activation [43]. Sickness behavior is notably characterized by social withdrawal. Chronic immune activation, such as through long-term air pollution exposure, can exacerbate this behavior and potentially lead to symptoms of depression. Although the cause-effect relationships are far from clear, exposure to fine particulate matter or diesel exhaust can result in an increase in pro-inflammatory cytokines in the striatum and hippocampus of rodents [44–47]. Other changes have been reported as well: Gestational and lactational exposure to traffic-related particulate matter in rats resulted in reduced fractional anisotropy, a measure of white matter tracts, in the hippocampus [48]. Further, ozone exposure in rats was shown to increase neuronal activation in the bed nucleus of the stria terminalis and in the medial amygdala [49], as well as to cause damage and neuronal loss in the hippocampus [50]. Oxidative stress was observed in the hippocampus and amygdala of rats exposed pre- and postnatally to fine particulate matter and/or gaseous pollutants [51]. Additionally, air pollution exposure may affect social behavior by altering hippocampal neurogenesis [52–54].

#### Neurochemicals

There are many signaling pathways, acting on both central and peripheral structures, important in the regulation of social behavior. Some of the most well-studied include sex steroids, glucocorticoids, biogenic amines, and the neuropeptides oxytocin and vasopressin [55]. Air pollution is composed of many endocrine disrupting compounds that can perturb these neuroendocrine systems [24, 56].

*Sex steroid hormones* – such as androgens, estrogens, and progestogens – have organizational and activational effects on brain and behavior [57], both of which can be perturbed by pollutant exposure [58]. During critical stages of pre- and postnatal development, testosterone binds to androgen receptors and, after aromatization to estradiol, estrogen receptors. The larger relative amounts of circulating testosterone in males derived from the testes results in signaling cascades that both masculinize and defeminize the developing brain. These organizational effects of steroid hormones on the brain are necessary for the expression of sexually dimorphic social behavior in adulthood [59]. Exposure to endocrine disrupting chemicals during critical periods of brain development can disrupt normal brain organization, as these compounds can act on both androgen and estrogen receptors, even at very low doses [22, 60]. Sex steroid hormones and their receptors also respond via activational effects to social stimuli over both immediate and longer-term timescales to modulate behavior [61]. Within minutes to seconds, steroid hormones can affect behavior via the activation of membrane-bound receptors. Within hours to days, steroid hormones modulate behavior via the activation of nuclear hormone receptors, leading to the transcriptional regulation of target genes. For example, under the ‘challenge hypothesis’ androgens transiently increase in response to social challenges in some social systems [62, 63], including in humans [64].

*Potential link to air pollution exposure*: Many known or suspected endocrine disrupting chemicals adhere to diesel exhaust particles (e.g., polycyclic aromatic hydrocarbons, heavy metals) and to particulate matter in general [65], thus diesel exhaust can interact with sex steroid hormone pathways via the release of these adsorbed chemicals, from direct effects of the particles themselves, or via exposure to gaseous components such as nitrogen dioxide. Diesel exhaust inhalation exposure has been shown to disrupt testosterone biosynthesis in rats [66], as well as alter serum testosterone levels following prenatal exposure [67]. Further, postnatal inhalation exposure to ultrafine particulate matter can reduce serum testosterone levels in mice [68]. Lastly, diesel exhaust secondary organic aerosol exposure reduces levels of estrogen receptor-alpha in the hypothalamus of mice [69, 70].

*Glucocorticoids* – another class of steroid hormones – are secreted from the adrenal glands following activation of the hypothalamic–pituitary–adrenal axis. Short-term activation of this axis results in an adaptive stress response that mobilizes energy reserves in the body (e.g., during agonistic social interactions). Responsiveness of the hypothalamic–pituitary–adrenal axis is influenced by social status, such as position in a dominance hierarchy [71], as well as by social factors during development [72].

*Potential link to air pollution exposure:* Exposures to ozone or particulate matter result in transient activation of the hypothalamic–pituitary–adrenal axis (via activation of sensory nerves in the respiratory tract or inflammation) and a subsequent increase in circulating levels of glucocorticoids [73, 74]. Notably, because social stressors can also dysregulate the hypothalamic–pituitary–adrenal axis, there may be synergistic effects between these stressors and air pollution exposure that further modify glucocorticoid signaling [75].

*Biogenic amines* (including serotonin and catecholamines such as epinephrine, norepinephrine, and dopamine) are metabolically synthesized in many different tissues, including the brain [76], where they act as neuromodulators. For example, dopaminergic signaling is essential for encoding the salience and rewarding properties of social stimuli and mediates social context-dependent behavior by changing the motivational state [77, 78]. Dopamine may also serve a critical role in linking social behavior and neuro-immune effects [79]. Serotonin (aka 5-hydroxytryptamine, 5-HT) is another biogenic amine with a role in regulating social behavior. Serotonin is often inversely related to aggressive behavior, particularly expression of the 5-HT_1A_ and 5-HT_1B_ receptor subtypes [80].

*Potential link to air pollution exposure*: Exposure to diesel exhaust particles can disrupt dopamine neuron function in vitro [81, 82]. In mice, prenatal inhalation exposure to diesel exhaust results in decreased levels of dopamine metabolites in the striatum and in increased levels of dopamine and its metabolites in the amygdala [83], as well as reduced dopamine turnover in the striatum [84] and increased dopamine levels in the nucleus accumbens [67]. Postnatal inhalation exposure to traffic-derived ultrafine particulate matter increases dopamine turnover in the hippocampus in female mice [47]. In addition, rats exposed to ozone show a decreased number of dopamine neurons in the substantia nigra [85]. Ozone exposure in rats decreased expression of serotonin receptor subtypes 5-HT_1A_, 5-HT_1B_, and 5-HT_4,_ increased 5-HT_2C_ in the hippocampus (a response partly regulated by glucocorticoids) [86], and reduced 5-HT levels in the hypothalamus [87].

*Neuropeptides* – small proteineous molecules produced in neurons by the processing of a genetically encoded precursor molecule – often act as neuromodulators. The nonapeptides oxytocin, vasopressin, and their non-mammalian homologs are exceptionally well studied for their role in mediating social behavior, in particular social attachment, recognition, affiliation, and parental behavior [88, 89]. These effects are highly conserved across species [90]. In addition, several links have been made between oxytocin signaling and autism spectrum disorder. In two different mice models, mutations in human autism risk genes resulted in impaired oxytocin signaling and autistic-like behavior [91, 92].

*Potential link to air pollution exposure*: Pre- and postnatal exposure in rats to fine particulate matter and/or gaseous pollutants results in decreased oxytocin receptor expression in the amygdala and hippocampus [51]. In mice, adult exposure to diesel exhaust secondary organic aerosols results in reduced gene expression of the oxytocin receptor in the hypothalamus [69]. In addition, there is growing evidence that oxytocin and vasopressin pathways are perturbed by endocrine disrupting chemicals [93], many of which are present in outdoor air.

### Effects of air pollutants on social behavior

As formalized in the developmental origins of adult disease hypothesis, exposure to environmental stressors during critical windows can affect disease susceptibility later in life [94]. In addition to potential perturbations during development, social behavior can also be affected by exposures later in life. Epidemiological studies have examined the effects of air pollution on social behavior-related outcomes during development and in adults, and experimental studies have begun to provide support for these effects. Thus far, these experimental studies are limited to rodent model systems, results of which are briefly summarized below.

#### Developmental exposures

Autism spectrum disorders are a group of disabilities that arise during development and affect social interactions and communication throughout adolescence and adulthood. Both genetic and environmental factors contribute to their etiology [95]. Several recent systematic reviews and meta-analyses have assessed the epidemiologic evidence base for the effects of air pollution exposure on autism spectrum disorders [96–98]. While noting limitations in the available evidence, each of these reviews identified an association between prenatal exposure to fine particulate matter and diagnoses of autism spectrum disorders. Furthermore, a separate study reported an association between social responsiveness in children and exposure to polycyclic aromatic hydrocarbons during prenatal development [17]. In general, based on the available studies described below, experimental animal studies have reported concordant results – inhalation exposure to air pollutants during development, including particulate matter, consistently affects aspects of social behavior in rodent models. These effects are often observed in males but not females, suggesting a sex bias in susceptibility. Diagnoses of several neurodevelopmental disorders, including autism spectrum disorders, are more prevalent in males. There is a wide range of likely mechanisms underlying this sex specificity, including disruption of androgenic signaling pathways during development and early maternal immune activation, both of which may arise from air pollutant exposure [99, 100].

In two studies, exposure to fine particulate matter during pre/postnatal development resulted in impaired social behavior in male but not female mice [46, 101]. In addition, treatment reduced social approach and preference for novel conspecifics, but did not affect social recognition [46, 101]. In a separate study, postnatal exposure to traffic-derived ultrafine particulate matter resulted in reduced social novelty preference in male but not female mice, which is likely related to abnormal testosterone levels during development [68]. In male rats, pre- and postnatal exposure to traffic-derived particulate matter reduced levels of social play, allogrooming, and nest building performance [48]. Finally, pre- and postnatal exposure to a mixture of fine particulate matter and gaseous pollutants impaired social novelty preference in rats [51]. Adult mice exposed pre- and postnatally to diesel exhaust showed reduced social interactions, decreased social novelty preference, and failed to habituate to a social odor [70, 102]. However, a similar study of diesel exhaust particles reported null results [103], potentially suggesting an important contribution of the gaseous component [104]. Finally, male mice prenatally exposed to diesel exhaust showed increased social isolation-induced territorial aggression [67]. The specific mechanisms through which developmental air pollutant exposures affect social behavior, with increased susceptibility in males, remain unclear but likely arise from interactions between the endocrine and immune systems [100].

#### Adult exposures

Several epidemiological studies have suggested that short-term changes in air pollutant exposure can affect human behavior. For example, increased levels of fine particulate matter and ozone were found to associate with heightened levels of violent, but not non-violent, crime [105, 106], a potential effect on aggressive behavior. In addition, a recent systematic review and meta-analysis reported that short-term exposure to nitrogen dioxide, but not to ozone, sulfur dioxide, or particulate matter, was positively associated with depression (which includes social withdrawal and isolation) [107]. A different meta-analysis found an association between long-term exposure (> 6 months) to fine particulate matter and depression [108]. Both reviews have pointed to the limitations in the evidence and suggest that more high-quality studies are needed to assess the effects of air pollution on mental health outcomes.

Few experimental studies have assessed effects of adult inhalation exposure to air pollutants on social behavior. Mice dams exposed to secondary organic aerosols from diesel exhaust showed decreased maternal performance [69]. In addition, there is evidence from mice that adult exposure to ozone, sulfur dioxide, or heavy metals from vehicle dust can inhibit aggressive behavior [109–111] and that ozone exposure can impair social interactions more generally [112]. Importantly, air pollution could also affect social behavior in adult animals by impairing sensory perception. For example, in species where chemical signals, such as pheromones, are important for mate attraction or social aggregation, chemical reactions with air pollution (oxidation from ozone, in particular) could shorten the distance signals can travel or impair receptor binding [113].

### Emerging research areas

There are several emerging and shared research areas within both the study of social behavior and in air pollution toxicology. For example, the role of the microbiome has become prominent. Microbes interact with their host through a variety of signaling pathways, including via the production of microbial-associated molecular patterns, neurotransmitters, hormones, metabolites, and immune cells. Host microbes are necessary for neurodevelopment, impact the central nervous system throughout adulthood, and have been implicated in social behavior [114–116]. Air pollution exposure appears to disrupt the microbiome, potentially via inflammation and altered epithelial cell permeability in the gut [117, 118]. The interaction between microbes and air pollution has the potential to bidirectionally impact social behavior, yet directly linking alterations in the microbiome to function and host health is challenging. Another important research area is in neuroepigenetics. Regulation of the chromatin of neurons affects behavior, and this regulation can be affected by environmental exposures [119]. In addition, environmental exposures can result in transgenerational epigenetic modifications with impacts on behavior [120, 121], though the link between air pollutants and social behavior has not yet been explicitly made. More broadly, emerging approaches toward the study of environmental exposures and neuropsychiatric diseases have been recently outlined [122].

### Translational potential

**Fig. 2:**
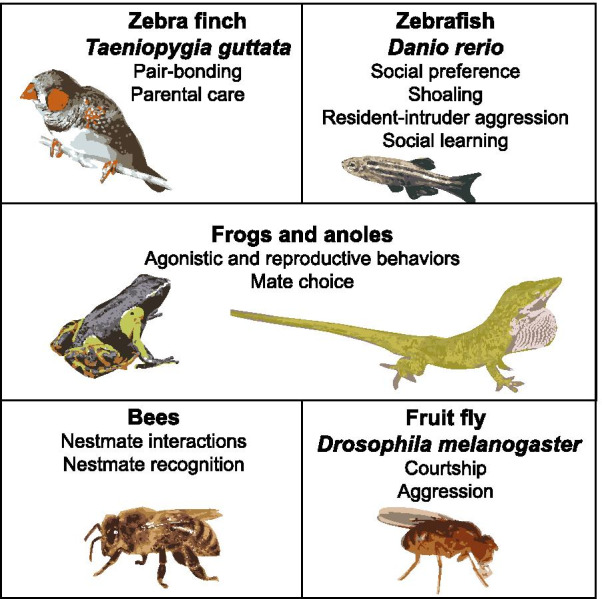
Complementary model systems in which to examine the effects of exposure to air pollution on social behavior

While important as model systems, laboratory rodents are limited in their behavioral repertoire. In addition to the benefits of a comparative approach (discussed below), examining the effects of air pollution exposure on diverse species allows an analysis of effects on a broad spectrum of social behaviors and their neurobiological mechanisms. Here, we highlight zebra finch, zebrafish, frogs and anoles, bees, and fruit flies as complementary model systems (Fig. 2). For most of these species, there is a full suite of genomic and molecular tools available that have allowed them to serve as model systems in social neuroscience [123, 124]. Employing these species in studies on the effects of air pollutants on social behavior can serve as a bridge between reductionist laboratory experiments and field-based ecotoxicology assays [125]. For example, while these species are used as models in the laboratory, they can also be collected in the wild, allowing for a realistic profiling of their exposome to characterize cumulative exposure [126]. Integrating the metabolome, proteome, transcriptome, and epigenome with behavior assays following both field and laboratory-based exposures can provide a systems-level view of the effects of inhaled pollutants [126]. An important aspect to these models, however, is their lack of, or distinct pulmonary systems compared to mammals. As the lungs are the primary target for air pollution, it will be critical to identify how different respiratory systems respond to air pollutant exposure and to clearly define the translational potential between humans and non-mammalian model systems.

#### Zebra finch

As canaries in the coal mine, birds have highly efficient respiratory systems that make them more sensitive to air pollutants compared to many mammalian species [127]. In addition, entire flocks can be exposed to environmentally-relevant mixtures of air pollutants under controlled conditions [128]. One of the most studied birds in social neuroscience is the zebra finch, *Taeniopygia guttata*. The zebra finch is monogamous, allowing for the study of the effects of air pollutants on pair-bonding and parental care behaviors in both males and females. Given evidence that the molecular underpinnings of monogamy are in part highly conserved across vertebrates [129], many neuromolecular mechanisms affected in zebra finch are likely shared with humans. Thus far, the limited number of studies available that employ zebra finch in toxicology primarily focus on dietary exposure and reproductive endpoints [130].

#### Zebrafish

The zebrafish, *Danio rerio*, may be a less obvious model system for studying air pollutant exposure compared to birds and cannot capture effects of inhalation, but nonetheless it holds great promise as a model system. In one study, zebrafish were used to examine effects of solubilized fine particulate matter on locomotor behavior [131]. This study and others suggest that the skin response in larval zebrafish can be predictive of mammalian lung epithelial responses [132]. Zebrafish are widely used as a model throughout biomedical science and specifically in the study of brain disorders [133–135]. In addition, they are being employed as a model to study the mechanistic basis of social behavior [136, 137]. Many assays have been developed to examine social behavior in zebrafish, including tests of social preference, shoaling, resident-intruder aggression, and social learning [138], including the use of sophisticated high-throughput video tracking methods [139, 140]. While zebrafish social behavior is not yet frequently examined in toxicity assays of air pollution mixtures, several studies have examined the effects of endocrine disrupting compounds [141, 142] and pharmaceuticals [139, 143] on social behavior.

#### Frogs and Anoles

Amphibians may be particularly sensitive to air pollutants given their potential for increased respiratory exposure due to gas exchange across the skin. Physiological effects of ozone exposure have been investigated in amphibians and reptiles, but to our knowledge no studies have examined effects on social behavior. Female mate choice assays, which are very robust in frogs, could be employed to examine whether air pollutants interfere with the hormonal modulation of behavior [144, 145]. In addition, Anolis lizards are amenable to assays of agonistic and reproductive behaviors [146, 147]. Both frogs and anoles are frequently used as model systems to examine the mechanistic basis of social behavior [147–150].

#### Bees

Honeybees, *Apis mellifera*, are highly social, living in colonies of thousands of individuals with distinct roles within the group. Honeybees have a long history as a unique model in neuroscience to study olfactory learning, and more recently as a model to study cognition [151]. Despite differences in neural architecture, the deep evolutionary conservation of social behavior genes makes mechanisms regulating behavior in bees relevant to humans [152, 153]. For example, similarities were found between autism-related genes in humans and socially unresponsive honeybees [154]. Honeybees have been used as biomonitors of air pollutants, and a limited number of studies have examined pollutant effects on olfactory learning and memory [155]. Effects on social behavior from air pollutants have yet to be investigated, however a recent study found an association between particulate matter air pollution and flower visitation, expression of stress-related genes, and survival in the Giant Asian honeybee, *Apis dorsata* [156]. Given the ability to track and monitor individual interactions at high spatial and temporal resolution [157], honeybees could become a powerful model system to examine effects of air pollution. Similarly, bumblebees, *Bombus sp.* species, share many of the advantages of honeybees as a model system but with significantly smaller colonies. In addition, standardized microcolonies of bumblebees can be studied under defined laboratory conditions, allowing for large sample size and replication capacities [158].

#### Fruit flies

The fruit fly, *Drosophila melanogaster*, is a classic genetic model system with an unparalleled molecular-genetic toolkit, as well as methods for high-throughput phenotyping of social behavior, such as courtship [159] and aggressive behavior [160]. The effects of indoor air pollutants on gene expression and locomotor behavior have been examined [161], suggesting this model is highly amenable to high-throughput testing of the effects of air pollutants on social behavior. Specifically, inhalation exposure to toluene or formaldehyde induced gene expression changes similar to those reported in mammals, affecting pathways related to stress and immune responses.

## Discussion

Great strides are being made in new approach methodologies in risk assessment and hazard identification, such as integrating in vitro and in silico approaches, and in -omics and high-throughput screening technologies. These new methods can produce a vast amount of mechanistic data for environmental chemicals, but without behavioral data it is challenging to assess the adversity of the outcomes. Thus, it has been suggested that behavioral outcomes be used as an organizing principle in neurotoxicology [162, 163]. While behavioral studies are critical to understanding the context of mechanistic effects from pollutant exposure, there is also a long-standing push to replace, reduce, or refine animal use in research. For example, the U.S. EPA recently called for an elimination of all mammal study requests and funding by 2035. To make progress toward the goal of limiting animal research, an improved understanding of the degree to which adverse effects and mode of action of air pollutant exposures are conserved across animals is needed. We suggest that this could be gained by a comparative and translational approach that expands both the species studied and the behavioral assays employed.

While research in laboratory rodents has driven much of our understanding of neuroscience and neurotoxicology, there are numerous potential advantages to complementing this body of knowledge by the study of more evolutionarily diverse species [123, 124, 164–166]. First, a phylogenetic comparative approach utilizing species across animal lineages that range in evolutionary distance to one another allows for hypothesis testing [20, 152, 167]. Further, clearly defining the conserved initiating events and mode of action though which air pollutant exposure may lead to adverse social-behavioral effects can help the field move toward more predictive frameworks [168, 169], an important initiative given that many more air pollutants and mixtures of pollutants exist than can be tested by behavioral assays. Finally, an additional benefit to studying the effects of air pollutants on social behavior in a broader range of species is that it can provide insight into possible causes of wildlife population declines worldwide. For example, though the mechanisms are unknown, ambient ozone concentrations have been associated with declines in bird populations in the U.S. [170].

Much progress has been made toward understanding the neural and molecular basis of social behavior across a wide range of species. More often than not, this research has shown that the mechanisms regulating social behavior are highly conserved across vertebrates and even beyond. However, this insight has not yet been widely integrated in applied research. There is a great opportunity to apply perspectives from evolutionary neuroscience and genetics toward the study of the behavioral and neurodevelopmental effects of environmental chemicals, and specifically toward examining the effects of air pollutants on social behavior. In addition, in humans, the adverse effects of air pollutants result from interactions among multiple stressors, many of which are non-chemical and psychosocial in nature (e.g., socioeconomic disparities, lack of family stability) [74, 171, 172]. There are opportunities within the model systems described above to simulate social stress, such as through dominance hierarchies, and to investigate the interactive effects from air pollutants.

## Conclusions

Given the overlap between conserved mechanisms of social behavior and alterations from air pollutants, such as modulation of the dopaminergic reward system, air pollution has the potential to impact a wide array of social behaviors in both humans and wildlife. Moving forward, it will be important to develop a framework that spans beyond neurodevelopmental and neuropsychiatric disorders to investigate effects of air pollutants on social behavior in a wider range of species, brain regions, and neurochemical pathways. In general, increased collaboration between neuroscientists, geneticists, and environmental health scientists has the potential to further all fields, in addition to benefitting public health [122].

## Data Availability

Not applicable.
